# Supplementary material for: Radiographic characteristics of impacted teeth: A retrospective study of 2199 radiograph
Source: PLoS One. 2025 Feb 13;20(2):e0316749. doi: 10.1371/journal.pone.0316749 (PMC11825008; doi:10.1371/journal.pone.0316749)
Supplement: Attachment — Submitted filename: PLOS Respond to reviewer Impaction.docx [file pone.0316749.s001.docx]

**Respond to Reviewer**

Re: **“Radiographic Characteristics of Impacted Teeth: A Retrospective Study of 2199 Radiograph”**

We really appreciate the reviewers’ time and efforts to help improve our manuscript.

For **Reviewer #1**:

Thank you for your comments. Please find the response for each point, beside highlighting it in the revised version of the manuscript.

1) Mention the power of the study. How was the sample size calculated.

The required sample size was calculated for prevalence and therefore is reported in this format with confidence level rather than power.

Added to the text (Material and methods). 377 or more research subjects are needed to have a confidence level of 95% that the real value is within ±5% of the measured value.

2) Mention the clinical significance of the study.

Added to the text (introduction).

3) Mention the mean age of Males and females separately.

Added to the results.

Mean age for female is 27.2 & mean age for male is 29.3.

4) Grammatical errors need to be addressed.

Done

For **Reviewer #2**:

Thank you for your valid opinion. We appreciated your suggestion. We considered the impacted canine in more details in our paper (highlighted) and we used the reference provided. We elaborated more in the result about the impacted canine. We have also updated the introduction as suggested.

Best regards,

Corresponding Author

**Arwa Ubaid Alsaggaf**, BDS, CAGS, MClinDent, MPros RCS

Assistant Professor and Prosthodontist, Department of Oral and Maxillofacial Surgery, Faculty of Dentistry, Umm Al-Qura University, 2373  [Al-Abdiyyah](https://maps.google.com/?q=(21.331949066820737,%2039.94123578071594)), Makkah, Saudi Arabia.

Mobile: +966554373717, Email: [ausaggaf@uqu.edu.sa](mailto:ausaggaf@uqu.edu.sa)
